# Correction: Modified TCA/acetone precipitation of plant proteins for proteomic analysis

**DOI:** 10.1371/journal.pone.0211612

**Published:** 2019-01-25

**Authors:** Liangjie Niu, Hang Zhang, Zhaokun Wu, Yibo Wang, Hui Liu, Xiaolin Wu, Wei Wang

S1–S3 Figs are incorrect. S1 Fig should be the 2D gels of maize embryos. The images for S2 and S3 Figs were incorrectly duplicate. Please see the corrected S1–S3 Figs below.

## Supporting information

S1 Fig Comparison of 2DE protein profiles of maize embryo proteins extracted using two methods. Shown were two independent experiments. Left panel: the modified TCA/acetone precipitation. Right panel: the classical TCA/acetone precipitation. Spots with increased abundance are indicated in red. About 800 μg of proteins were resolved in pH 4–7 (linear) strip by IEF and then in 12.5% gel by SDS-PAGE. Proteins were visualized using CBB.
(TIF)

S2 Fig Comparison of 2DE protein profiles of maize root proteins extracted using two methods. Shown were three independent experiments. Left panel: the modified TCA/acetone precipitation. Right panel: the classical TCA/acetone precipitation. Spots with increased abundance are indicated in red. About 800 μg of proteins were resolved in pH 4–7 (linear) strip by IEF and then in 12.5% gel by SDS-PAGE. Proteins were visualized using CBB.
(TIF)

S3 Fig Comparison of 2DE protein profiles of maize leaf proteins extracted using two methods. Shown were three independent experiments. Left panel: the modified TCA/acetone precipitation. Right panel: the classical TCA/acetone precipitation. Spots with increased abundance are indicated in red. About 800 μg of proteins were resolved in pH 4–7 (linear) strip by IEF and then in 12.5% gel by SDS-PAGE. Proteins were visualized using CBB.
(TIF)
