# Supplementary figures and images for: Modified TCA/acetone precipitation of plant proteins for proteomic analysis
Source: PLoS One. 2018 Dec 17;13(12):e0202238. doi: 10.1371/journal.pone.0202238 (PMC6296544; doi:10.1371/journal.pone.0202238)

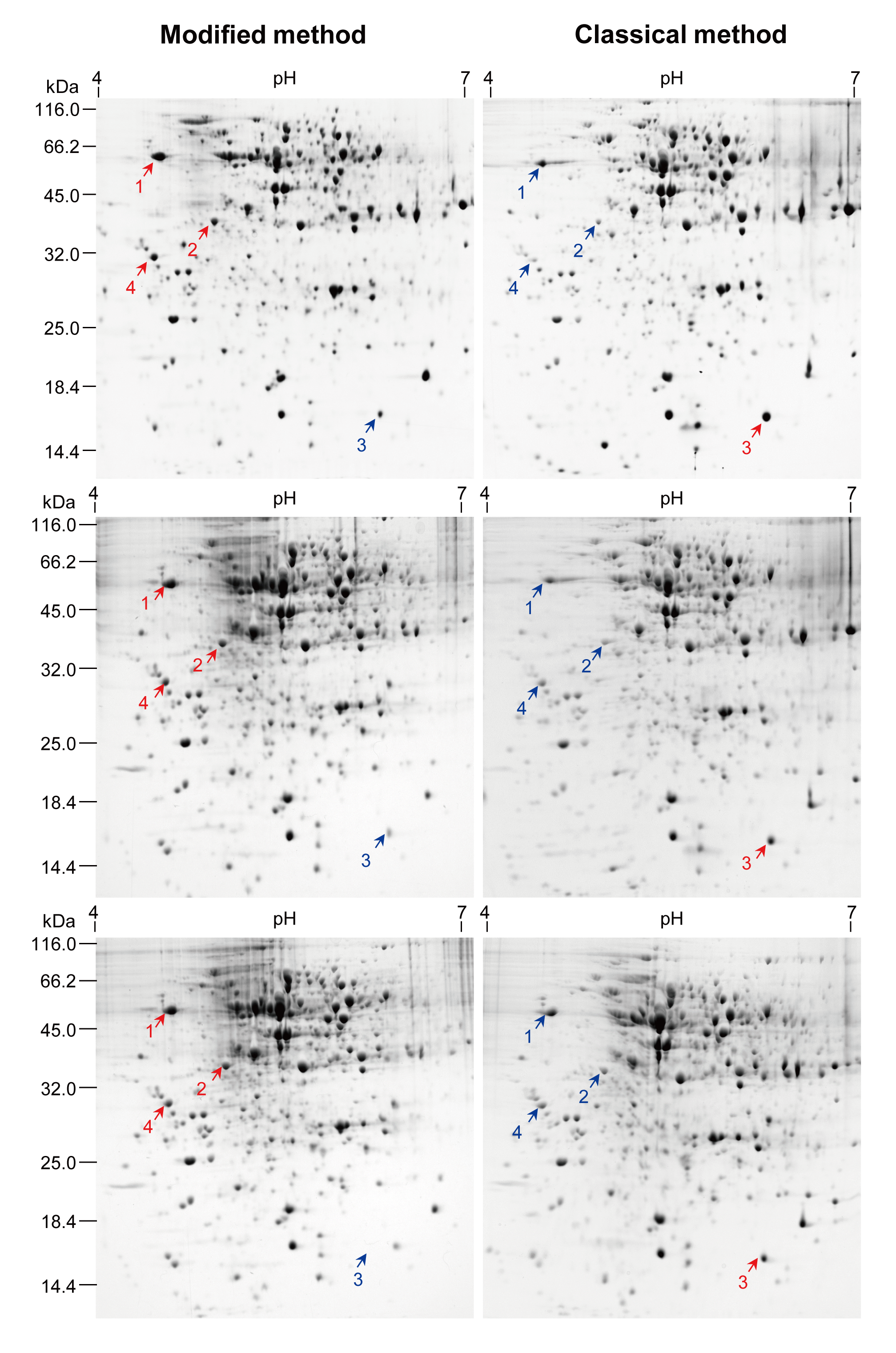

Supplement: S1 Fig — Shown were two independent experiments. Left panel: the modified TCA/acetone precipitation. Right panel: the classical TCA/acetone precipitation. Spots with increased abundance are indicated in red. About 800 μg of proteins were resolved in pH 4–7 (linear) strip by IEF and then in 12.5% gel by SDS-PAGE. Proteins were visualized using CBB. (TIF) [file pone.0202238.s001.tif]

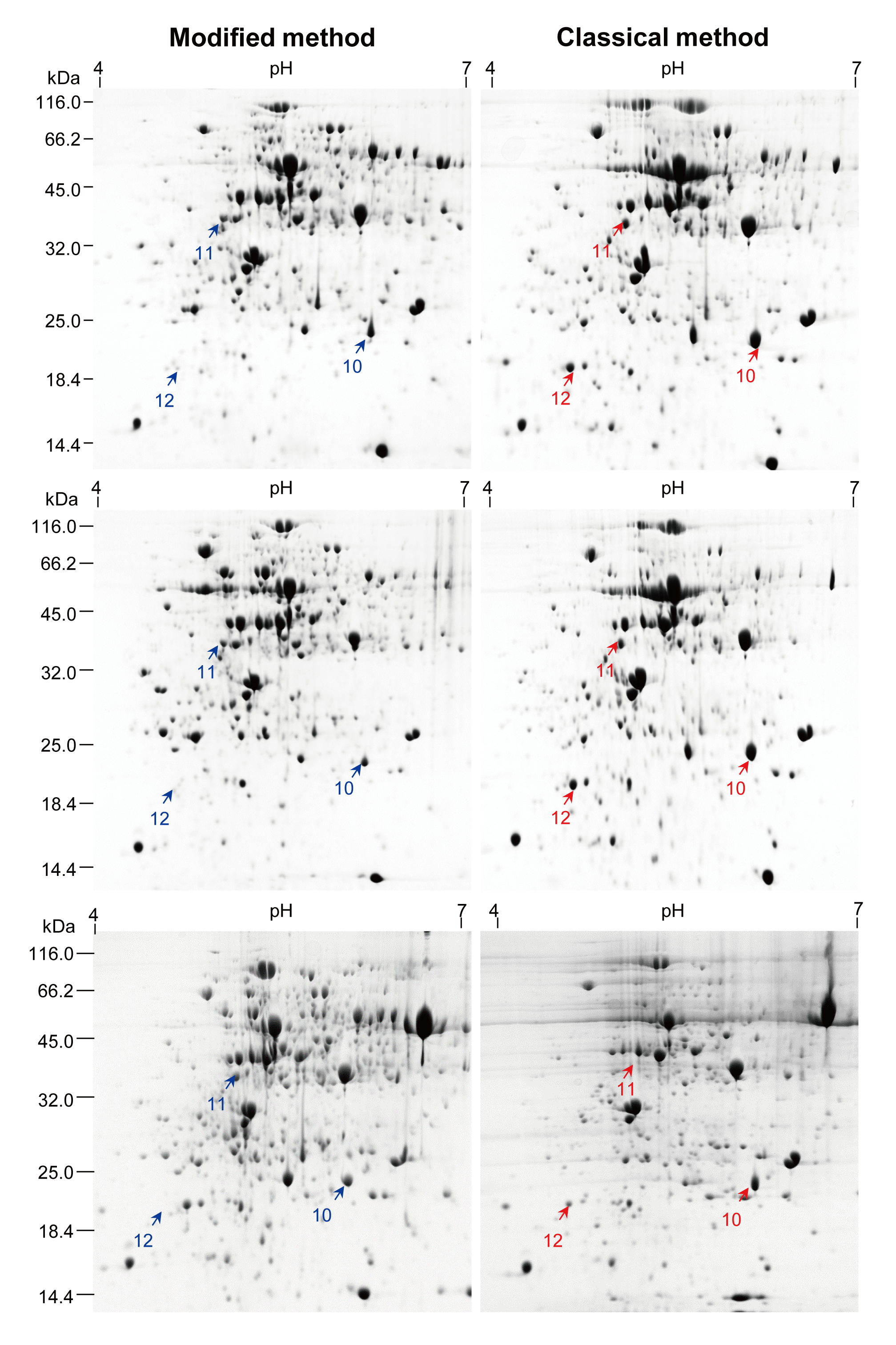

Supplement: S2 Fig — Shown were three independent experiments. Left panel: the modified TCA/acetone precipitation. Right panel: the classical TCA/acetone precipitation. Spots with increased abundance are indicated in red. About 800 μg of proteins were resolved in pH 4–7 (linear) strip by IEF and then in 12.5% gel by SDS-PAGE. Proteins were visualized using CBB. (TIF) [file pone.0202238.s002.tif]

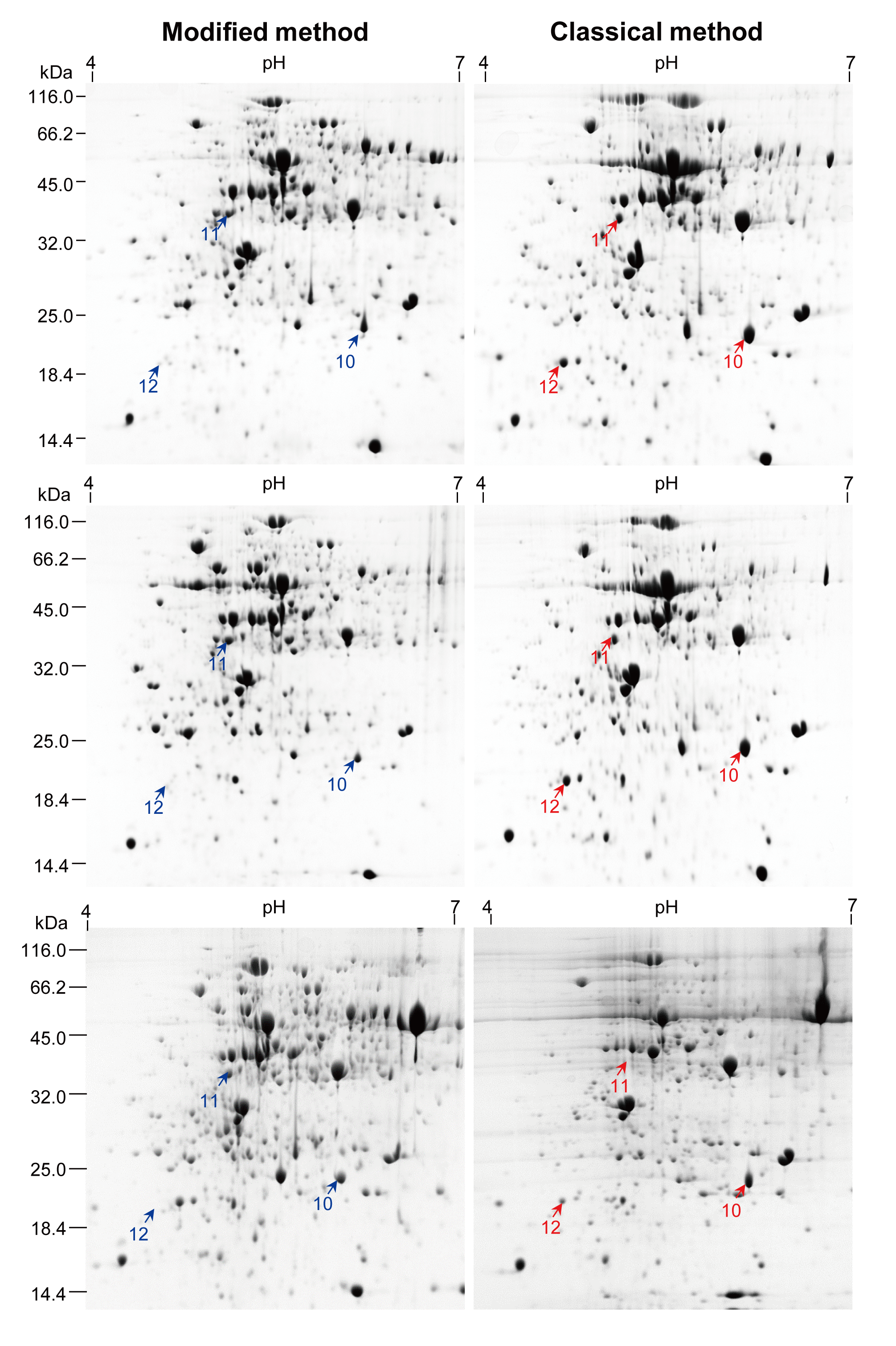

Supplement: S3 Fig — Shown were three independent experiments. Left panel: the modified TCA/acetone precipitation. Right panel: the classical TCA/acetone precipitation. Spots with increased abundance are indicated in red. About 800 μg of proteins were resolved in pH 4–7 (linear) strip by IEF and then in 12.5% gel by SDS-PAGE. Proteins were visualized using CBB. (TIF) [file pone.0202238.s003.tif]

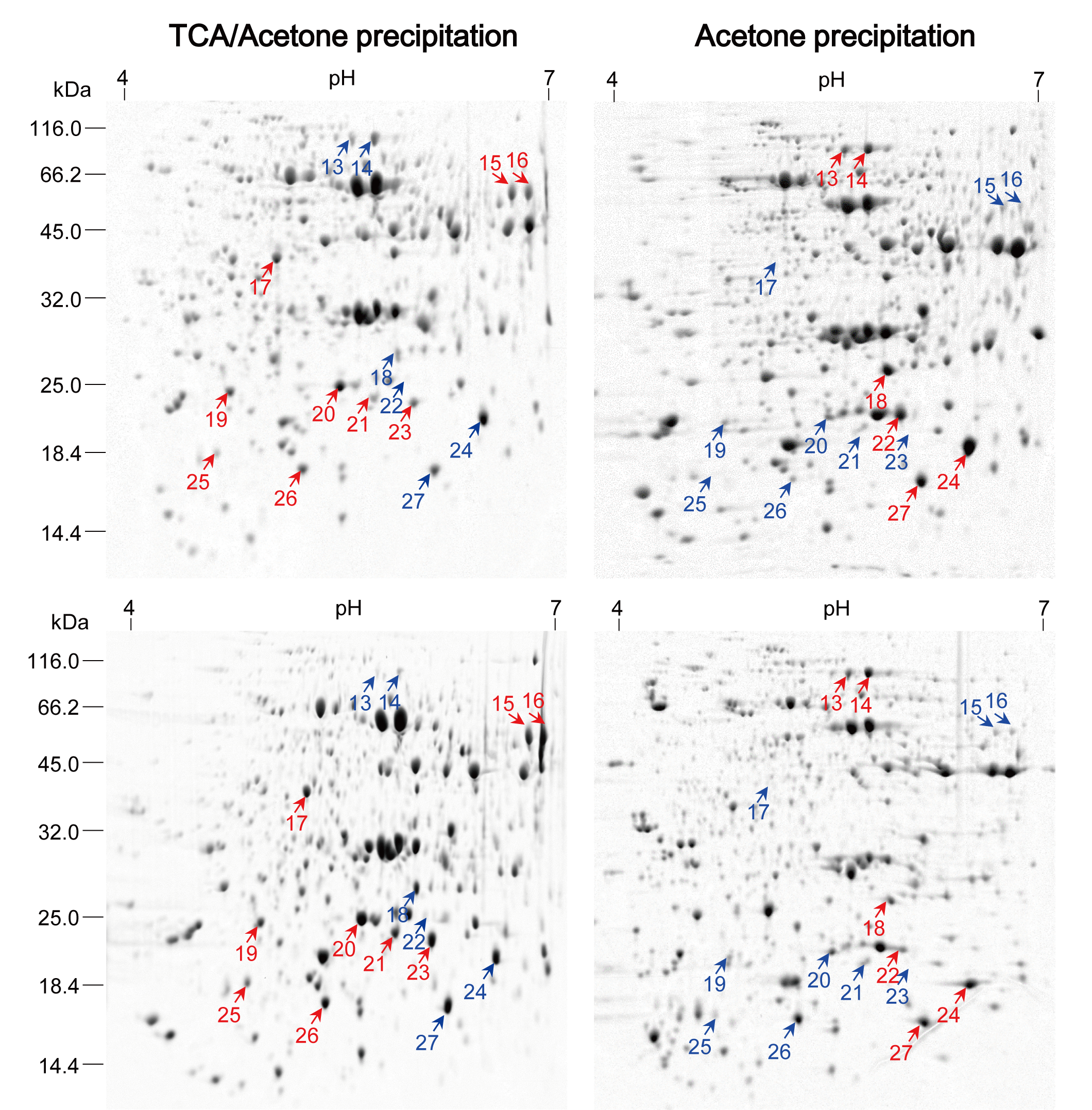

Supplement: S4 Fig — Shown are two independent experiments. Left panel: the modified TCA/acetone precipitation. Right panel: acetone precipitation. About 800 μg of proteins were resolved in pH 4–7 (linear) strip by IEF and then in 12.5% gel by SDS-PAGE. Protein was visualized using colloidal CBB. (TIF) [file pone.0202238.s004.tif]
