# Supplementary figures and images for: Correction: Modified TCA/acetone precipitation of plant proteins for proteomic analysis
Source: PLoS One. 2019 Jan 25;14(1):e0211612. doi: 10.1371/journal.pone.0211612 (PMC6347226; doi:10.1371/journal.pone.0211612)

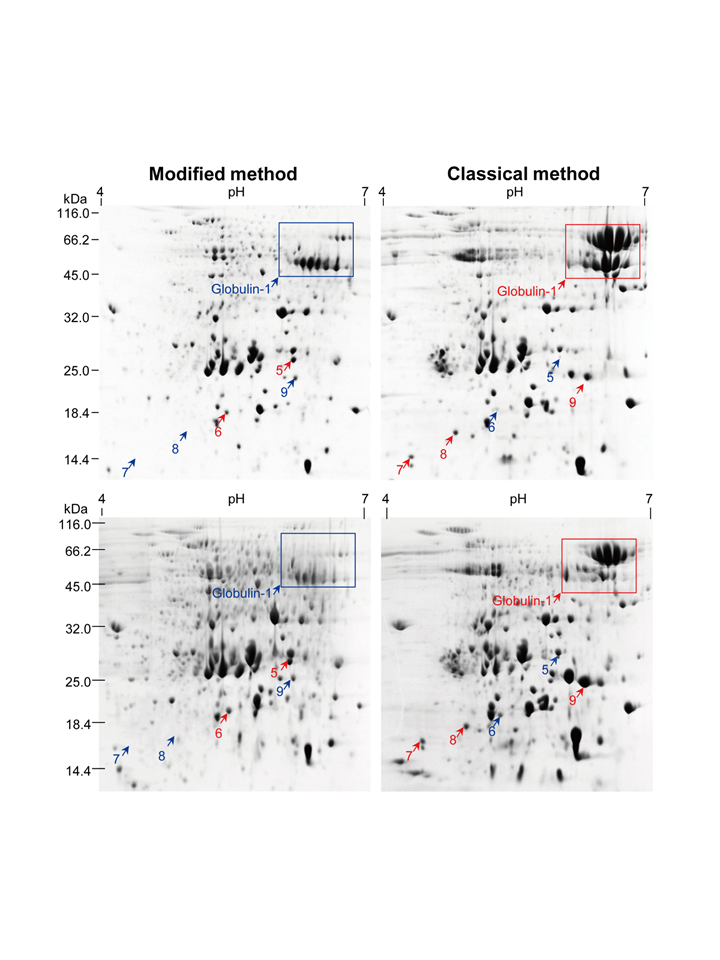

Supplement: S1 Fig — Shown were two independent experiments. Left panel: the modified TCA/acetone precipitation. Right panel: the classical TCA/acetone precipitation. Spots with increased abundance are indicated in red. About 800 μg of proteins were resolved in pH 4–7 (linear) strip by IEF and then in 12.5% gel by SDS-PAGE. Proteins were visualized using CBB. (TIF) [file pone.0211612.s001.tif]

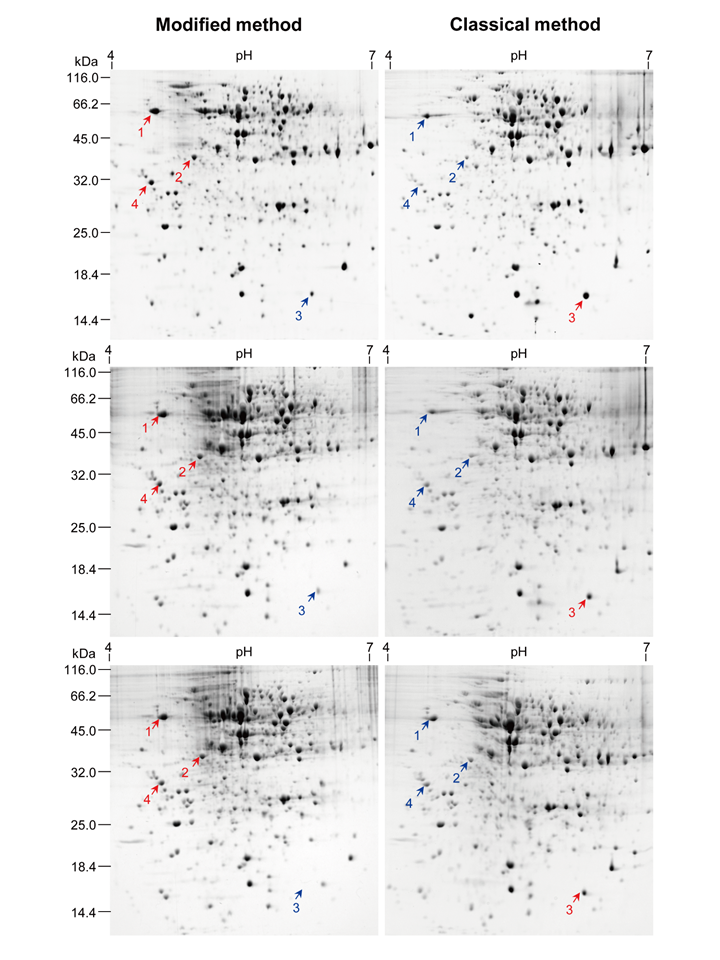

Supplement: S2 Fig — Shown were three independent experiments. Left panel: the modified TCA/acetone precipitation. Right panel: the classical TCA/acetone precipitation. Spots with increased abundance are indicated in red. About 800 μg of proteins were resolved in pH 4–7 (linear) strip by IEF and then in 12.5% gel by SDS-PAGE. Proteins were visualized using CBB. (TIF) [file pone.0211612.s002.tif]

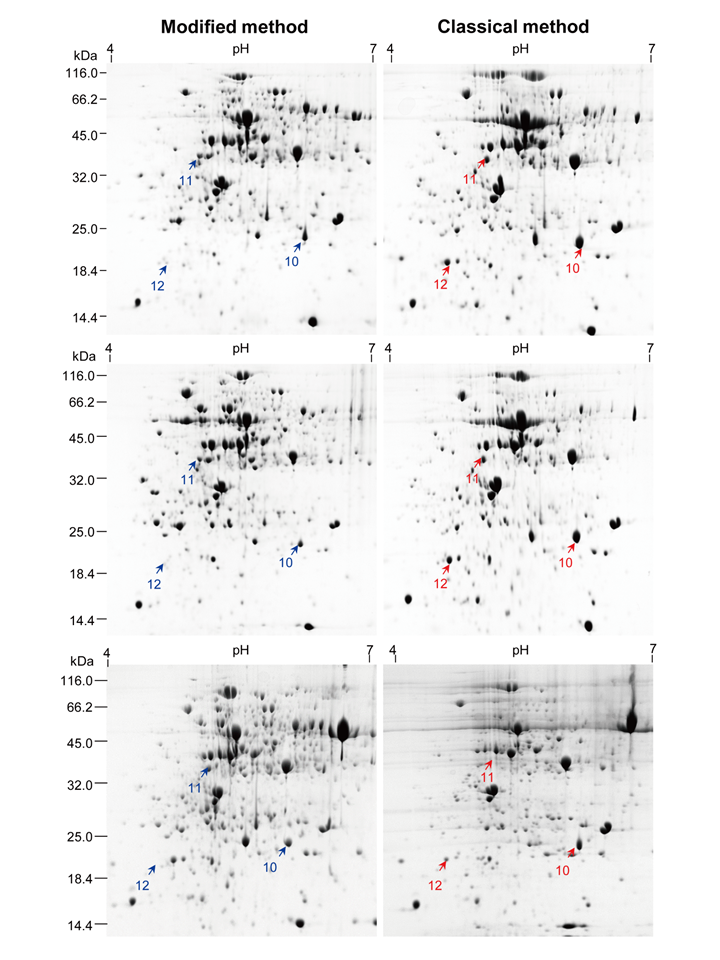

Supplement: S3 Fig — Shown were three independent experiments. Left panel: the modified TCA/acetone precipitation. Right panel: the classical TCA/acetone precipitation. Spots with increased abundance are indicated in red. About 800 μg of proteins were resolved in pH 4–7 (linear) strip by IEF and then in 12.5% gel by SDS-PAGE. Proteins were visualized using CBB. (TIF) [file pone.0211612.s003.tif]
